# Developing more participatory and accountable institutions for health: identifying health system research priorities for the Sustainable Development Goal-era

**DOI:** 10.1093/heapol/czy079

**Published:** 2018-09-20

**Authors:** K Scott, N Jessani, M Qiu, S Bennett

**Affiliations:** 1 Health Systems Program, Department of International Health, Johns Hopkins Bloomberg School of Public Health, 615 N. Wolf Street, Baltimore, MA, USA; 2 Department of Health Behavior and Society, Johns Hopkins Bloomberg School of Public Health, 615 N. Wolf Street, Baltimore, MA, USA

**Keywords:** Health policy and systems research, research priorities, evidence-based policy, evidence-to-action, Sustainable Development Goals, social accountability, governance

## Abstract

Health policy and systems research (HPSR) is vital to guiding global institutions, funders, policymakers, activists and implementers in developing and enacting strategies to achieve the Sustainable Development Goals. We undertook a multi-stage participatory process to identify priority research questions relevant to improving accountability within health systems. We conducted interviews (*n* = 54) and focus group discussions (*n* = 2) with policymakers from international and national bodies (ministries of health, other government agencies and technical support institutions) across the WHO regions. Respondents were asked to reflect on challenges and current policy discussions related to health systems accountability, and to identify their pressing research needs. We also conducted an overview of reviews (*n* = 34) to determine the current status of knowledge on health systems accountability and to identify any gaps. We extracted research questions from the policymaker interviews and focus groups (70 questions) and from the overview of reviews (112 questions), and synthesized these into 36 overarching questions. Using the online platform Co-Digital, we invited researchers from around the world to refine and then rank the questions according to research importance. The questions that emerged amongst the top priorities focused on political factors that mediate the adoption or effectiveness of accountability initiatives, processes and incentives that facilitate the acceptability of accountability mechanisms among frontline healthcare providers, and the national governance reforms and contexts that enhance provider accountability. The process revealed different underlying conceptions of social accountability and how best to promote it, with some researchers and policymakers focusing on specific interventions and others embracing a more systems-oriented approach to understanding accountability, the multiple forms that it can take, how these interact with each other and the importance of power and underlying social relations. The findings from this exercise identify HPSR funding priorities and future areas for evidence production and policy engagement.

**Key Messages**

- The Sustainable Development Goals call for the creation of equitable and strong institutions, which includes developing more participatory and accountable health systems.
- To channel research funding and effort towards areas of greatest importance for supporting health system accountability, it is vital to set research priorities, and to do so in a participatory manner that engages relevant stakeholders.
- Our multi-stage priority setting process involving academics and policy actors enabled the identification and ranking of priority research questions, which can guide funders and researchers in the coming years.
- Seven of the top ten questions focus on context, process and implementation factors that mediate or influence accountability initiatives, suggesting that experts strongly position accountability improvements as embedded in the broader political and health system context.

## Introduction

Rude or indifferent treatment at health facilities, medicine stock outs, absent personnel, demand for informal payment, unresponsive management and unfulfilled promises—accountability failures in the health sector are well documented in academic literature and popular media, and experienced firsthand by millions of people every day. These failures violate patient dignity and rights, and lead to preventable morbidity and mortality.

Global attention to the need for improved accountability within government services has grown since the World Bank introduced the concept of ‘good governance’ in 1989 (World Bank, 1989) and the World Health Organization drew attention to ‘stewardship’ in the 2000 World Health Report (WHO, 2000; Gaventa and Barrett, 2010; Pyone *et al.*, 2017). In 2015, the United Nations adopted the Sustainable Development Goals (SDGs), which enshrined the value of accountability in goal 16 on ‘peace, justice and strong institutions’. This goal encompasses several targets focused on accountability, including to substantially reduce corruption and bribery (16.5), develop effective, accountable and transparent institutions (16.6), ensure responsive, inclusive, participatory and representative decision-making (16.7) and promote and enforce non-discriminatory laws and policies (16.9B) (United Nations, 2016).

The SDGs present a unique global challenge and opportunity, including for those working to improve health outcomes and strengthen health systems. While more difficult to conceptualize than bounded, narrow or disease-focused goals, the comprehensiveness and complexity of the SDGs create an opportunity for innovative thinking and action that recognizes the interconnectedness between health and other social and environmental systems, including issues of accountability within the citizen–state relationship.

There are several forms of accountability, including social, political and bureaucratic. Social accountability—which maps onto the concept of ‘demand side’ accountability—involves any act or strategy through which citizens seek to hold the state to account (O’Meally, 2013). Social accountability seeks to both bolster citizen engagement and strengthen the responsiveness of the state to citizens (Brinkerhoff, 2004). Political accountability seeks to improve the responsiveness of elected officials through voting and engaging elected representatives (Cleary *et al.*, 2013). Bureaucratic accountability involves oversight of public services, actors and processes by those internal to the system (public sector employees and elected officials) through mechanisms such as routine supervision or internal financial audits (Cleary *et al.*, 2013). Bureaucratic accountability maps onto the concept of ‘supply side’ accountability, which includes public sector reforms and internal checks and balances (anti-corruption bureaus, open budgeting, legislative oversight capacity-building, grievance redress mechanisms, etc.).

Efforts to develop more participatory and accountable institutions for health involve negotiating power between three core actors: citizens, healthcare providers and policymakers or government officials (World Bank, 2003). Research on accountability has often distinguished between a ‘short route’ and ‘long route’ (World Bank, 2003; Baez-Camargo, 2011). This theory suggests that citizens can influence health care through the ‘short route’ of direct engagement with the providers through applying ‘voice’, i.e. pressure using mechanisms such as community score cards and health committees, or being able and willing to ‘exit’, i.e. seek health outside the public sector (Paul, 1991). The ‘long route’ places the policymakers as mediators in the citizen–provider relationship, where citizens delegate authority to political representatives, who in turn govern bureaucracies by appointing policymakers, who in turn manage frontline providers. However, recent work by Fox (2015) suggests that the long route/short route dichotomy breaks down upon analysis, since ‘short route’ relationships between citizens and providers are embedded in governance issues all the way up bureaucratic state structure. Similarly, ‘supply side’ and ‘demand side’ accountability mechanisms interact and blur when applied, e.g. when citizen pressure (demand side) leads to the creation or bolstering of bureaucratic accountability processes (supply side).

To achieve the SDGs and strengthen health systems accountability, policymakers, funders, activists and implementers must collaborate with researchers to generate and use evidence on how best to approach this complex issue, channel resources, structure programmes and policies, bolster implementation and measure change. Research priority setting exercises focus finite funding and bolster support for research on issues that maximize public health benefit and are most pertinent for informing change (Mcgregor *et al.*, 2014). To this end, we engaged in a participatory, multi-stage research priority setting exercise. This exercise identified policymaker needs for information and evidence as well as gaps in academic knowledge to develop a list of potential research questions. The questions were subsequently refined and ranked, allowing us to produce a list of priority research questions that can support the development of more participatory and accountable institutions for health in the SDG-era. This paper describes the process undertaken to develop and rank these priority research questions, and presents the results.

## Methodology

The priority setting process involved four stages: an overview of reviews on accountability in health; consultations with policymakers through interviews and focus group discussions; extraction and synthesis of research questions from the review and policymaker consultation; refinement and ranking by researchers using Co-Digital, an online platform.

### Overview of reviews on social accountability in health

We conducted an overview of reviews to identify knowledge gaps and map the breadth of the field, including the types of research questions asked, research settings, theoretical contributions and evidence. We focused on pre-existing reviews rather than primary research studies to pragmatically and rapidly map the large body of literature on social accountability in health.

#### Search strategy

The first author, in collaboration with an academic librarian, developed comprehensive searches to identify reviews on three sub-topics within health system accountability and participation: health committees, score cards and other forms of local health system governance and accountability (including decentralization). Two electronic databases, Scopus and Pubmed, were searched using a combination of controlled vocabulary and keyword terms for each of the concepts (see Supplementary Annexure S1 for further details). All duplicates were deleted and unique references were exported to Microsoft Excel for screening. We included all literature up to 1 December 2016. The first author also examined references of included articles for additional reviews and received suggestions from experts.

#### Study selection and criteria

Articles were included if they were (1) reviews (systematic and non-systematic reviews were included), (2) English language, (3) included significant content on accountability (i.e. mechanisms to bolster citizen engagement and state or service provider responsiveness), (4) applied to national or sub-national health systems or health programmes and (5) focused on or included content on low- and middle-income countries (LMICs). In applying these inclusion criteria, we excluded reviews on: the accountability of researchers to communities (e.g. community participation in ethics review boards for clinical trials); patient participation in their own health care (e.g. diabetes self-care) or community participation in health service provision (e.g. bed net distribution); health governance at the global level; health system decentralization in terms of patient care outcomes rather than citizen power and oversight (e.g. linking decentralized care and increased access to antiretroviral treatment, but without discussing accountability); improving the social consciousness and ethical behaviour of health professionals through curriculum changes (because they did not engage with citizen power) and civic engagement without any health focus (e.g. studies on the accountability of education or forestry sectors). All articles presenting primary research were also excluded. The full texts of included articles were retrieved and screened again to ensure they met the inclusion criteria.

During the screening process, we identified two broad categories of review paper: those that reviewed the empirical literature on interventions to improve accountability, and those that reviewed conceptual understandings of accountability. We focused on the empirical review papers for our data extraction and synthesis, and also drew from the conceptual papers where relevant to frame the broader conceptual mapping of the literature.

#### Data extraction

The following data from the included articles were extracted: (1) metadata; (2) overview of the review; (3) findings on civic engagement and local accountability including health committees; (4) findings on decentralization; (5) findings on score cards; (6) findings on promoting transparency about the performance of local health systems; (7) finding on internal (bureaucratic) accountability; (8) conclusions and reflections on research quality and (9) knowledge gaps or outstanding research questions (for the detailed data extraction form, see Supplementary Annexure S2). These categories enabled us to capture the wide range of possible approaches to social accountability by including findings on specific accountability interventions or processes that we were already seeking (committees, score cards, decentralization) with categories flexible enough to capture accountability processes we had not anticipated.

#### Data synthesis

Data were synthesized to identify the types of interventions used to improve accountability, the evidence of effectiveness, conceptual debates and challenges and knowledge gaps or research questions identified in the reviews.

### Consultations with policymakers

The overview of reviews was complemented by in depth interviews (IDIs) and focus group discussions (FGDs) with senior level policymakers from around the world as well as senior staff from large multi-lateral or bilateral organizations and NGOs. Three sources were consulted for identification of participants: (a) participant lists at two major global conferences attended by the study team: Health Systems Global 2016 (Vancouver, Canada), and the Prince Mahidol Awards Conference 2017 (Bangkok, Thailand), (b) recommendations from colleagues at the World Health Organization’s Alliance for Health Policy and Systems Research (AHPSR) and (c) through relationships with colleagues based in select countries, namely India, South Africa, Lebanon and Argentina. We did not attempt to sample policymakers in a manner that would have enabled country-level saturation or cross-country comparisons—this would have required a sample many times larger than the scope of this work, and would not have furthered our goal of broadly understanding policymaker priorities globally; instead, policymaker respondents were understood as key informants, each bringing unique perspectives to the issue that could only be analysed holistically to inform overarching and broad policymaker perspectives.

Participants were contacted via email, by phone or in person. The majority of the IDIs were conducted face-to-face except in instances where geographic location posed a challenge. In such cases, interviews were conducted over the phone. FGDs were held in two countries—Bahrain and Jordan—so as to elicit reflections from policymakers in the Eastern Mediterranean Region, which were underrepresented in the IDIs.

The IDIs explored policymaker perceptions regarding health systems challenges anticipated with respect to meeting the SDGs in their contexts, and policy changes being considered to mitigate these challenges. The IDIs then focused specifically on three themes, of which ‘developing more participatory and accountable institutions for health’ was one. Specific questions around challenges, policy considerations and information and evidence needs for this theme were then explored more deeply.

IDIs and FGDs were audio-recorded with participant permission. Reflections were captured in detailed notes by the interviewer/facilitator. Any responses collected in Arabic, French or Spanish were translated into English for analysis. These notes were subsequently used to populate a matrix of results allowing the team to use a framework analysis approach for data analysis (Gale *et al.*, 2013). The framework approach enabled us to easily compare how the policymakers (in rows) commented on the various content areas (in columns) to identify trends, gaps and differences in their responses, enabling rapid synthesis and easy identification of comments relevant to pressing research priorities.

The study was deemed ‘non-human subjects research’ by the Johns Hopkins Bloomberg School of Public Health Institutional Review Board.

### Identifying research questions

#### Extraction of research questions from the overview of reviews

All research questions and knowledge gaps identified in the reviews were extracted into an excel file. In cases where research questions were not articulated directly but instead were implicit within broader reflection by review authors on the state of evidence, the research team synthesized and re-phrased the remarks into questions. These questions were then migrated into a separate Google spreadsheet, with one question per cell and its source mapped in the adjacent cell. In instances where identical or very similar questions were found across articles, all possible sources were indicated. These research questions were then grouped into thematic areas.

#### Extraction of research questions from the policymaker consultations

Responses from the IDIs and FGDs with policymakers on research needs related to health system accountability were extracted and matched, where relevant, to the thematic areas from the overview of reviews and entered into the Google spreadsheet. In instances where new questions or ideas arose, additional thematic areas were created to complement those from the reviews.

#### Synthesis of questions

To synthesize the questions from the various sources into distinct questions for ranking, we collapsed similar questions into overarching ones. For example, we developed the question ‘How do you harmonize various sources of data (internal, external, community-led, audits, etc.) in order for information to be a driver for accountability?’ by synthesizing four similar questions from the reviews and one question from an interview. In some cases, the overarching question synthesized up to 10 questions with a similar intent. In most cases the overarching question was a synthesis of three or fewer. Several overarching questions re-phrased only one question from the source material.

### Refinement and ranking of research questions

Co-Digital (www.codigital.com), an online collaboration platform, was used to further revise and rank the research questions developed by the study team. We selected this online platform because it allows for a large number of participants to propose edits and vote on them in real-time. Our participant consultation process through the Co-Digital platform spanned two phases. During phase 1 (14–23 August 2017), participants were invited to refine the research questions by proposing editorial changes. In phase 2 (28 August–6 September 2017), they were asked to rank the revised options so as to produce a list of priority questions. Each participant received a summary of the overview of reviews and an excel spreadsheet with a collated list of research questions from the review and policymaker consultations in advance of the Co-Digital process.

Based on the overview of reviews, and recent conferences and seminars that had focused on accountability for health, we identified 47 experts in the field, who also reflected diverse geographies and disciplines, as well as spanning low-, middle- and high-income countries. Thirty-two of the 47 accepted the invitation to participate in the online refinement and ranking exercise.

Of the 32 individuals who agreed to participate, 25 contributed to 81 unique edits and cast a total 225 votes for the edits during this phase. The study team reviewed the final generation of each research question from this exercise and incorporated all edits that remained consistent with the original intent of research question. In instances where this did not happen, we reverted to the original or a previous generation of the question. A final set of research questions were then uploaded to Co-Digital for the second phase.

During phase 2, the same 32 individuals were re-invited to cast votes for priority research questions. Through several rounds of this pairwise ranking process, high priority research questions were determined. The final scores for the ranking were unweighted, and calculated based on the number of times a research question won when competing head-to-head for votes with another research question. In this phase, a total of 25 individuals participated, and they cast a total of 491 votes. At the end of this exercise, a final ranking of the research questions was shared with the participants, and they were requested to share their feedback on the process and results.

## Results

### Overview of review of reviews

**Figure 1.**
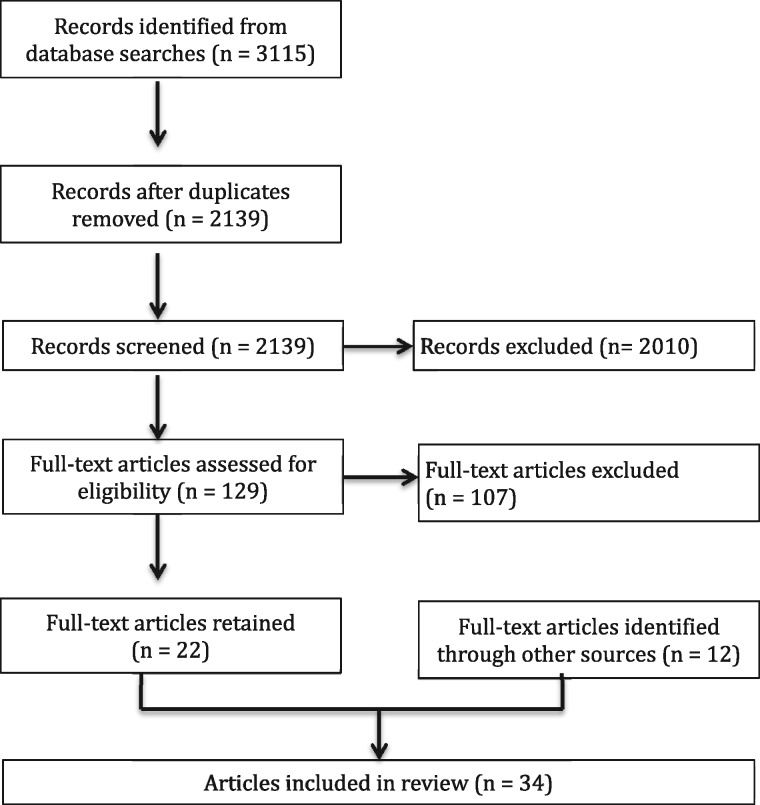
Diagram of search results for the review of reviews.

The searches resulted in a total of 3115 records. We pooled the results and deleted duplicates, leaving 2139 records to screen. Based on the title and abstract review, we identified 129 articles of potential relevance. Full text versions of these 129 articles were retrieved and reviewed against the inclusion and exclusion criteria. Ultimately, 22 review articles that focused on empirical research were identified through the database search and screening. An additional 12 resources were found through examining the references of included reviews and through expert inputs, for a total of 34 included review articles (Figure 1).

There is a large body of literature on community participation in health systems more generally, some of which maps onto the social accountability literature. Community participation can range from community members providing services to their peers, such as when community health workers distribute medicines, which is not a form of accountability, to community members participating in health facility monitoring, which is a form of accountability. We included reviews on community participation that included findings on accountability (Atkinson *et al.*, 2011; Rifkin, 2014; George *et al.*, 2015a), to ensure that the large body of community participation literature remained visible in this review of reviews.

In addition to the 34 review articles identified, which draw from empirical research to synthesize findings from the literature, we also identified numerous reports by agencies and research groups including the World Bank, Oxfam, Abdul Latif Jameel Poverty Action Lab and the Institute of Development Studies that examine aspects of social accountability (Paul, 1991; Ringold *et al.*, 2009; Gaventa and Barrett, 2010; Olken and Pande, 2011; Camargo and Jacobs, 2013; Lynch *et al.*, 2013; O’Meally, 2013; Rodden and Wibbels, 2013; Hoffman, 2014; McGinn and Lipsky, 2015; Wild *et al.*, 2015; Bradshaw *et al.*, 2016) and recent theoretical papers discussing conceptual issues within the field of social accountability (Gomez, 2011; Abimbola *et al.*, 2014; Barbazza and Tello, 2014; Joshi, 2014; Belle and Mayhew, 2016; Abimbola *et al.*, 2017; Pyone *et al.*, 2017). While these reports and theoretical papers informed the review, they were not synthesized through the data extraction framework as they either lacked clear methods or did not systematically present empirical evidence.

Of the 34 articles focused on in the overview of reviews, five had a global focus and 29 focused on LMICs (Table 1). Some reviews focused on specific accountability interventions, such as health committees (McCoy *et al.*, 2012; George *et al.*, 2015b), social audits (Pattinson *et al.*, 2009) and report cards (McNamara, 2006; Gullo *et al.*, 2016). Others looked at systems-level change through decentralization (Talukder *et al.*, 2008; Mitchell and Bossert, 2010; Cobos Muñoz *et al.*, 2017) or efforts to reduce corruption (Vian, 2008; Hanna *et al.*, 2011; Ciccone *et al.*, 2014; Gaitonde *et al.*, 2016; Molina *et al.*, 2016). Most reviews sought to identify interventions that influence accountability and thus discussed a range of interrelated interventions and outcomes ranging from health system responsiveness to maternal mortality.

**Table 1.** Overview of the reviews

Review	Type of review (as identified by the authors)	Country focus	Theme	Review focus
Atkinson *et al.* (2011)	Systematic review	Global	Community participation and infectious disease control and elimination	What factors influence community participation in infectious disease control or eradication?
Berlan and Shiffman (2012)	Synthesis	LMIC	Health care provider accountability	What factors shape health provider accountability to consumers, and what interventions can enhance responsiveness?
Ciccone *et al.* (2014)	Literature review	LMIC	Governance and health	What is the relationship between good governance (e.g. rule of law, government effectiveness and corruption perception; strong institutions) and health?
Cleary *et al.* (2013)	Descriptive literature review	LMIC	Governance mechanisms between actors (government, patients, providers)	How can accountability relations among the three categories of health systems actors (government, patients and providers) at the primary care level support governance?
Cobos Muñoz *et al.* (2017)	Systematic review	LMIC	Decentralization	What effects goes decentralization have on LMIC health systems?
Dieleman *et al.* (2011)	Review of case studies	LMIC	Health workforce policy implementation	How have governance issues influenced HRH policy development? What governance strategies have been used, successfully or not, to improve health workforce policy implementation in LMICs?
Dieleman *et al.* (2009)	Realist review	LMIC	Health worker performance	What interventions improve health workers' performance in LMICs?
Fox (2015)	Meta-analysis	LMIC	Impact of social accountability interventions	What are the limits of current social accountability conceptual frameworks? What impact do social accountability strategies have? How can we better analyse the dynamics of social accountability strategies?
Gaitonde *et al.* (2016)	Systematic review	Global	Corruption	What strategies have been used and how effective are they to reduce corruption in the health sector?
Gaventa and Barrett (2010)	Qualitative synthesis (meta-case study analysis)	LMIC	Outcomes of citizen engagement	What is the impact of participation on improved democratic and developmental outcomes?
George *et al.* (2015a)	Narrative review	LMIC	Health systems research	What is the extent, nature and quality of community participation in health systems intervention research?
George *et al.* (2015b)	Narrative review	LMIC	Health committees	What contextual factors influence health committees?
Gilson *et al.* (2014)	Interpretive synthesis	LMIC	Health worker performance	How do actors at the front line of health policy implementation exercise discretionary power, with what consequences and why?
Gullo *et al.* (2016)	Systematic review of project reports	LMIC	Community score cards	What has CARE’s experience of community score cards been across the variety of sectors and contexts, in terms of governance, service and development outcomes, as well as implementation challenges?
Hanna *et al.* (2011)	Narrative review	LMIC	Corruption	What types of policy levers are available to reduce corruption, and what have rigorous evaluations of these policies found?
Hoffman (2014)	Review of selected initiatives	LMIC	Maternal and child mortality	What are the common themes and methods used in three NGO-led social accountability initiatives seeking to improve health outcomes?
Holeman *et al.* (2016)	Scoping review	LMIC	Digital technology	What is the potential use and effect of mobile phones and other information and education technologies for health care accountability and governance in LMICs?
Joshi (2013)	Review	LMIC	Impact of transparency and accountability initiatives on service delivery (not just health)	Does increasing transparency or supporting social accountability initiatives lead to the desired outcomes? What are the assumed links through which these impacts are expected to occur?
Kalter *et al.* (2011)	Literature review	LMIC	Social autopsy	How widely and successfully has the social autopsy method been adopted?
Kaplan *et al.* (2013)	Literature review and assessment of 20 LMIC health systems	LMIC	Health workforce governance	How are LMICs progressing towards achieving the eight governance principles in their health workforces?
Lodenstein *et al.* (2016)	Realist review	LMIC	Provider responsiveness to accountability initiatives	How do social accountability initiatives, in different contexts, influence health provider responsiveness to citizens’ demands?
Lynch *et al.* (2013)	Systematic review	LMIC	Impact on service delivery	What is the evidence that the establishment or use of community accountability mechanisms and processes improves inclusive service delivery by governments, donors and NGOs to communities?
McCoy *et al.* (2012)	Systematic review	LMIC	Health committees	What is the evidence of effectiveness of village health committees?
McNamara (2006)	Review	LMIC^a^	Report cards	What evidence is there for the utility of report cards, and what are the design considerations that affect the how report card influence provider accountability?
Meads *et al.* (2017)	Systematic review	Global^b^	Collaborative governance	What are the categories for good collaborative governance?
Mitchell and Bossert (2010)	Review of evidence from 6 countries	LMIC^c^	Decentralization	What is the appropriate balance between centralization and decentralization of functions to improve health systems?
Molina *et al.* (2016)	Systematic review	LMIC	Community monitoring effect on corruption	What is the effectiveness of community monitoring interventions in reducing corruption?
Molyneux *et al.* (2012)	Review	LMIC	Community accountability at peripheral facilities	What are the mechanisms and outcomes of measures to enhance community accountability at peripheral health facilities?
Mubyazi and Hutton (2012)	Narrative review	LMIC	Community participation in health planning, resource allocation and service delivery	What factors influence the impact of community participation on health planning, resource allocation and service delivery?
Pattinson *et al.* (2009)	Systematic review	LMIC	Neonatal mortality audit, with meta analysis of impact on perinatal mortality	Does instituting a neonatal mortality audit improve quality of care in LMICs?
Rifkin (2014)	Review of systematic reviews	Global	Community participation and improved health outcomes	What are the links between community participation and health outcomes?
Salam *et al.* (2014)	Review of systematic reviews	Global	District level inputs to improve maternal and newborn health care	How effective are district level inputs to improve the quality fo care for maternal and newborn health
Talukder *et al.* (2008)	Four-country comparison	LMIC^d^	Health sector reform (including decentralization)	What lessons can we learn from health sector reforms?
Vian (2008)	Review	LMIC	Reducing corruption in the health sector	What are the causes of corruption in health systems and what interventions have been implemented to address it?

a Draws from HIC literature to discuss potential of report cards in LMICs.

b Draws lessons from Western Europe and South/Central America.

c Six countries: Bolivia, Chile, India, Pakistan, the Philippines and Uganda.

d Bangladesh, Pakistan, Indonesia and Tanzania.

#### Priority research questions

We identified 112 research questions from the reviews and grouped them into 15 thematic areas to help us identify overlapping questions. The thematic areas around which many questions clustered included calls for evidence of effectiveness, the need for comparison across contexts and timelines, and for better understanding of processes and incentives. The list of themes can be found in Supplementary Annexure S3.

### Consultations with policymakers

We conducted 54 interviews (47 of which included discussion on accountability), and two focus group discussions (both of which included discussion on accountability) across five WHO regions. The distribution of respondents identified and ultimately included can be found in Tables 2 and 3.

**Table 2.** IDI respondent summary by geographic region

WHO region	Respondents identified and invited	Respondents included	Respondents discussing social accountability	Interview language(s)
Africa Region	30	12	11	English
Region of the Americas	14	10	10	English, Spanish
South-East Region	18	14	12	English
European Region	0	0	0	NA
Eastern Mediterranean Region	5	4	3	English, Arabic, French
Western Pacific Region	15	8	7	English, Mandarin
Multi/Bi-Lateral Org/NGOs	7	6	4	English

**Table 3.** FGD respondent summary

FGD country	Respondents invited	Respondents included	FGD language
Bahrain	16	10	English
Jordan	17	17	Arabic

#### Policymaker identified research areas

Policymakers were concerned about a range of issues with respect to participatory and accountable institutions for health. The effectiveness and cost-effectiveness of some of the interventions related to social accountability appeared several times. Related to this was a desire to learn from countries that had already tried various models and approaches. For instance, policymakers in Bahrain wanted to learn about the impact of existing initiatives. Policymakers from Vanuatu, Uganda and the Caribbean wanted to learn what best practices exist, if there were benchmarks, what worked, what failed and how these learnings can be adapted to other contexts. Within-country adaptation was also recognized as important given the varying contexts: geographic (urban/rural settings), perspectives (gender/class), etc. For instance, a policymaker explained that Indian villages are very complex and divided by gender and caste. Since different groups would have different views on issues related to accountability, the policymaker emphasized the need for good research to sift out the core messages.

Implementation of good models was accompanied by queries about monitoring and evaluation of new initiatives. A policymaker from Kenya, e.g. indicated that they are currently trying to develop guidelines for social accountability and community scorecards but were unsure of how to monitor accountability. Similarly, an Indonesian policymaker wondered how to dovetail ongoing monitoring with independent evaluation of some of their modes of accountability such as a complaints system. In Zimbabwe, the discussions around monitoring were articulated from the perspective of empowerment and ownership. The Zimbabwean respondent wanted research to identify the kinds of training programmes for community members that are effective in supporting community monitoring and/or community engagement with the health sector. Similarly, policymakers from Bahrain and Ghana expressed a desire to know more about how to enhance public participation. In contrast, policymakers from countries such as India and Ghana as well as a multi lateral development organization expressed concerns not about creating space for public participation, but rather why existing mechanisms were not producing the desired results; more specifically, why communities in particular were not exercising their rights to demand more accountability.

Policymakers were also keen to learn about how to translate gains at the local level more widely. Scaling up interventions (such as scorecards) as well as processes (participatory mechanisms) to enhance social accountability was raised by representatives from bilateral development partner organizations and an independent think-tank, as well as by policymakers from countries including Bhutan, Pakistan, and Indonesia where decentralization is underway. In a similar vein, policymakers from India, Pakistan and Myanmar wondered whether there was a greater role for media and whether outreach and messaging needed to be tailored better for the desired stakeholders.

Integral to social accountability was a desire to enhance multi-stakeholder participation. A policymaker from Uganda noted that the biggest challenge is not having a culture of accountability, suggesting that Uganda needs to ‘fuel a generation that thinks about accountability’. This same policymaker called for greater community participation across a range of stakeholders including churches. This sentiment was echoed by policymakers from Vanuatu and Thailand who asked how to get all stakeholders, including community members, to engage on an equal platform and to participate at the same level.

This interest in engaging a range of stakeholders to enhance accountability linked to concerns about fostering multi-sectoral collaboration. For instance, one policymaker highlighted that in Indonesia there is the Supreme Audit Body, Ministry of the Civil Service, the Presidential Staff and Ombudsman, but it is unclear what role these various non-health-related agencies should play in the health sector. The role of academia was also questioned, with an Argentinian respondent asking how to involve social actors, mainly universities and research institutes, in the SDGs.

Innovation in data collection to inform more participatory forms of social accountability was discussed a few times. A representative of an independent think tank wanted to explore whether there are more innovative ways to collect information from patients in real time, and a policymaker from India wanted to see research on whether digital platforms such as phone apps and call centres that monitor pregnant women ‘are worth it’. A respondent from a bilateral development partner organization sought more research on simple tools that could help communities hold broader health systems accountable, including tools to help communities investigate unexplained deaths or ways for them to report on outbreak responses.

Data quality, integrity and use were important aspects of policymaker reflections. Several respondents, including from India and Uganda, were concerned about the duplication of data collection in the country and whether more efficient processes could be developed to collect and use data. Gaps between research and policy were noted across a range of topics, including on accountability. For instance, a respondent from Ghana noted that there is ‘a whole section of people presenting research findings on my country that I’m not aware of’ and sought mechanisms to bridge the gap between the implementers and the researchers.

Some policymakers noted that there were costs (financial, social and other) involved in social accountability initiatives and wondered about mechanisms to estimate the costs of new interventions and/or policies that enhance social accountability. These policymakers were also interested in mechanisms to measure the financial cost of compliance and to track inefficient use of funds.

#### Priority research questions from consultations with policymakers

We extracted 70 questions on accountability from the policymaker interviews and focus group discussions. These questions were added to the google spreadsheet already containing the 112 questions extracted from the reviews.

### Synthesis of research questions

The questions from the reviews and policymakers were synthesized into a final list of 36 research questions, available in Supplementary Annexure S4. Many questions from the consultations mapped onto similar questions from the reviews. For example Molina *et al.*’s (2016) review noted the need for research to answer the question ‘How does information-for-accountability diffuse among citizens’ social networks?’ and an Indian policymaker wondered about the potential of mobile phone use among youth to spread information about accountability issues.

The two synthesized questions that brought together the greatest number of individual questions from reviews and policymaker consultations focused on (1) the impact (expected and unexpected) or effectiveness of transparency and accountability interventions on various aspects of governance and health system performance and (2) the structures, processes and incentives that empower (or fail to empower) citizens to engage with accountability initiatives. Both were generated through a synthesis of 12 questions from reviews and six from policymaker consultations. Two questions were generated only from the policymaker interviews: social accountability in a context affected by conflict and refugee populations (Jordan FGD) and the alignment of global mechanisms for accountability with local initiatives (development partner organization). Nine questions were generated only from the reviews: the extent to which accountability initiatives can address issues across the macro, meso and micro levels; effectiveness across longer timeframes; impacts of interventions on the health workforce; how to facilitate the acceptability of accountability among senior policymakers and elites; improving theoretical models of accountability; understanding how the organizational environments of different health care facilities influences their accountability; contextual factors that trigger accountability activities and reasons for failed accountability interventions.

### Refinement and ranking of research questions

#### Participatory refinement to priority question wording in Co-Digital platform

Participant suggestions for refining the priority questions largely involved grammatical changes or the addition of further examples. For example, the original question ‘What processes and incentives (e.g. financial/non-financial, punitive/trust-building) facilitate the acceptability of accountability mechanisms among frontline healthcare providers?’ was refined in Co-Digital to add learning loops and peer review to the list of potential processes and incentives that could facilitate the acceptability of accountability mechanisms. Supplementary Annexure S4 lists the original questions, the revised questions and the number of unique revisions made to each original question. All questions were edited at least once and three questions underwent five rounds of editing, the maximum.

Several refinements sharpened the focus of research questions, including for the question that ended up ranked as the highest priority. The initial question ‘What factors (e.g. the discretionary power of health workers, media) mediate the adoption or effectiveness of accountability initiatives (e.g. digital technology, health committees, local media or more informal citizen actions)?’ was edited to specifically refer to *political* factors. Many refinements involved suggestions that a sub question on context be added, making it clear that for every area of research on accountability in health, attention to contextual enablers and challenges will be vital to deeply understanding the processes at hand and enabling analytic generalizability to other settings. We accepted all edits that had broad support from participants and did not deviate from the objective or original intention of the research question that emerged from review and/or policymaker consultations.

#### Final ranking of priority research questions

Participants in the final ranking were asked to consider which questions would have highest potential benefit or impact if researched, as well as the tractability of the research question and the extent to which answering the question would benefit poor and marginalized communities. We did not specify that the ranking should be based specifically on *public health* benefit or impact, instead leaving it open to the experts to prioritize research according to the type of benefit or impact they felt to be most relevant and appropriate. The final ranking exercise enabled us to order the 36 questions from highest to lowest priority (Supplementary Annexure S4). Table 4 presents the top 10 questions, along with final scores, which indicate the percentage of times a question was selected as higher priority when compared to another question.

**Table 4.** Top 10 ranked research questions on participatory and accountable institutions for health

Rank	Question	Final score
1	What political factors (e.g. the discretionary power of health providers, politicization of the health system and other political factors) mediate the adoption or effectiveness of accountability initiatives (e.g. digital technology, health committees, local media or more informal citizen actions)?	70%
2	What processes and incentives (e.g. financial/non-financial, punitive/trust-building, learning loops, peer review) facilitate the acceptability of accountability mechanisms among frontline healthcare providers?	69%
3	What reforms (e.g. decentralized budgeting, performance-based financing) in the governance of national health systems are most likely to enhance provider accountability to consumers and in what contexts?	68%
4	What mechanisms and contextual/historical factors enable or hinder various actors (MoH officials, lay and professional health workers themselves, clients and communities, civil society, private sector, religious groups providing healthcare) to interact productively to improve accountability and responsiveness?	68%
5	What conditions or factors are necessary to enable accountability initiatives to address issues at the macro (e.g. political social, cultural and economic environment), meso (e.g. organizational culture, incentives) and micro (e.g. individual ethics, rationalizations) levels?	63%
6	What are the impacts (expected and unexpected) of accountability interventions on the health workforce? (Attitudes, behaviour, practices, morale, decision-space, service provision, corruption, performance, etc.)	58%
7	What is the impact (expected and unexpected) or effectiveness of transparency and accountability interventions on various aspects of governance and health system performance (e.g. healthcare quality, service utilization, human resource management, corruption, participatory decision-making and citizen–state relationships within and beyond the health sector)?	58%
8	How can citizen monitoring and evaluation be effectively integrated into health system planning and implementation?	58%
9	What tools and design features (e.g. format, frequency of use, degree of standardization) can enhance the effectiveness of accountability initiatives, such as digital reporting tools, report cards, social audit tools/social autopsy tools, community report on outbreak responses?	58%
10	How do specific contexts (e.g. political environment, strength of democracy, social cohesion/heterogeneity, level of economic inequity, health system privatization) influence the potential for success/failure of particular types of accountability initiatives?	57%

The top ranked questions focus primarily on understanding processes and contextual factors that enable or hinder accountability (questions ranked #1, #2, #3, #4, #5 and #10). However, there is also one technical question on tools and design features (#9), and two measurement questions, that seek assessments of the impact of accountability interventions on the health workforce (#6) and on a range of aspects of governance and health system performance (#7).

Questions that ranked lowest were related to cost-effectiveness of different accountability mechanisms (#36), accountability in conflict-affected areas (#35), researcher accountability to communities and health systems (#34), circumstances that led to failed accountability interventions (#33) and the impact of accountability interventions on health and social outcomes (#32).

We examined the differences between the priority rankings, the number of questions from reviews that were integrated to generate the synthesized question, and the number of questions from the policymaker consultations that were integrated to generate the synthesized question. This assessment enabled an approximate comparison between the three sources of data on research needs with the strong caveat that results provide only a rough proxy for the importance that academic reviewers and policymakers assign to the various questions. For instance, a review may not highlight the need for future research on an issue because the authors are aware of another existing review already making a similar recommendation. A policymaker may not mention a certain research topic for reasons unrelated to the importance of the issue.

Nonetheless, our assessment found that 4 of the top 10 highest priority questions were generated from just one or zero policymaker questions (#2, #5, #6 and #8) and several top 10 questions also arose from two or fewer review questions (#3, #4 and #5). In contract, 3 of the top 10 highest priority questions had high convergence across the three sources: Question #7, on the impact of accountability interventions, was one of the most discussed questions in the reviews and policymaker consultations, arising from 12 review questions and six policymaker questions—the highest number of source questions; question #9, on tools and design features that can enhance the effectiveness of accountability initiatives, and question #10, on contextual influences, were derived from questions that arose frequently in the reviews, and were discussed fairly often by policymakers.

Many lower ranked questions were derived from few or no reviews and few or no policymaker comments (e.g. questions 25, 30, 31, 33, 34 and 35)—essentially, all three sources suggested that these questions require less emphasis. However, questions on cost-effectiveness (#36) and the impact of accountability initiatives on health and social outcomes (#32) were ranked quite low during the Co-Digital process, despite having been derived from a fairly high number of source questions from the reviews and policymaker consultations.

## Discussion

This priority setting exercise enabled the identification and ranking of 36 research questions on developing more participatory and accountable institutions for health from a review of reviews, policymaker consultations and a participatory refinement and ranking process. Seven of the top ten highest ranked questions focused on context, process and implementation factors that mediate or influence accountability initiatives, suggesting that experts strongly position accountability improvements as embedded in the broader political and health system context. Two focused on measuring outcomes (on health system performance and the health workforce) and one on identifying technical aspects of interventions that can improve their effectiveness.

Research questions that arose from a higher number of reviews tended to also be ranked higher priority. However, there was not a strong alignment between questions that policymakers mentioned multiple times and highly ranked research priorities. Similarly, several research questions that arose from a high number of policymaker consultations ranked below the top 10.

Our study did not gather data to fully explore potential divergence between the priority rankings by experts and the number of related questions integrated from reviews and policymakers. While there may be a mismatch between priority areas for research identified by authors of reviews on accountability, by policymakers working in LMIC health systems, and by the accountability experts who engaged in the ranking exercise, other explanations are worth exploring. Experts may have considered some research questions important but ranked them lower in priority because they were too difficult to answer or because answering them would not yield useful findings across contexts. Policymakers may not have mentioned some questions that were ranked as high priority by experts simply because they had not yet considered these issues or had not considered the role that research in the area could play in helping with policy and programming. In some cases, questions overlapped with one another and divergent rankings may have been more indicative of preferred syntax or salient examples, rather than meaningful distinctions. For instance, question #32 on evidence that accountability interventions improve health and social outcomes was of great interest to policymakers but was ranked towards the bottom of the priority list. The expert rankers may agree that evidence for the impact of accountability on health and social outcomes is of high priority but expressed this through their high ranking of similar questions: #6 on the impact of accountability interventions on the health workforce and #7 on governance and health system performance.

Further research and engagement with all three stakeholder groups (authors of reviews, policymakers and the accountability experts who engaged in the ranking exercise) would help understand the extent to which expert priorities varied from those of policymakers and academic reviewers. Researchers and funders using the priority rankings to guide future work should ensure buy-in from both academic and policymaker stakeholders from the onset of proposal development, to ensure that funded work is considered to be of high value to all relevant actors.

There is a large and rapidly growing body of research on accountability, which includes both empirical work on effectiveness and theoretical work on models, frameworks and theories. Nonetheless, there remains much work to be done in both areas, and in building shared conceptualization of accountability and how to approach it. The plethora of terms and models applied to this field indicates its richness and complexity but can also create confusion and reduce the ability for work to be compared or generalized across settings (Brinkerhoff, 2004; Barbazza and Tello, 2014). For example, while recent innovative research conceptualizes accountability as tactical and strategic (Fox, 2015), other conceptualizations highlight upward and downward reporting (Mitchell and Bossert, 2010), internal/bureaucratic and external pathways (Cleary *et al.*, 2013) or long route and short route mechanisms (World Bank, 2003; Baez-Camargo, 2011). While each conceptualization is valuable in its capacity to highlight different aspects of the phenomena, this diversity can also cause fragmentation and confusion, particularly when seeking to facilitate communication among researchers, funders, policy stakeholders and civil society.

When seeking information about how to improve accountability and how effective various efforts have been, the review of reviews and priority research questions identified in this project speak to the existence of divergence in how accountability is conceptualized. Some reviews and priority research questions focus more narrowly on specific interventions, e.g. asking whether score cards or health committees achieve a measurable impact on health outcomes, or how best to design tools for accountability. Other reviews and priority questions embrace a more systems-oriented approach to understanding accountability, the multiple forms that it can take, how these interact with each other and the importance of power and underlying social relations. This systems-oriented work highlights the need for multi-level, multi-pronged, politically engaged efforts that strengthen the citizen–state relationship. However, it remains unclear how to support these complex processes and what role single, bounded, interventions may play.

The field of health policy and systems seeks to integrate research and policymaking so that research findings can bolster evidence-informed policy and support implementation (WHO, 2007; Bennett *et al.*, 2018). Using a transparent process to set the research agenda and involving policymakers and researchers in the process sets a strong foundation for improved integration of research to policy to practice. To this end, our priority setting exercise brought together decision-makers and researchers from around the world and enabled them to identify priority areas for future research. The use of an online platform to revise and rank questions fostered engagement according to each participant’s availability at a lower financial, time and carbon cost than convening face-to-face meetings. By presenting a detailed description of the priority setting process here. All stakeholders are invited to engage with and build upon our methodology.

Several limitations must be considered. The scoping review, in an effort to rapidly map the breadth of the field, focused on published academic literature and thus did not account for the rich body of gray literature. The policymaker consultations engaged respondents working in health but some were new to their positions or had not had significant time and experience to develop strong understandings of accountability in their country’s health sector. This frequent turnover and transfer of policymakers must be kept in mind as researchers seek to engage policymakers throughout the research cycle. Furthermore, policymakers, when asked to express the challenges they faced in meeting the SDGs and the evidence or research that could help them, often articulated perspectives that were difficult to translate into research questions. We found that many of those who agreed to participate in the refining and ranking exercise were drawn from the health policy and systems research community, and thus attend many of the same conferences and webinars. While the HPSR field includes academics from diverse backgrounds, this concentration of participants from a similar intellectual pool may have resulted in the rankings being influenced by a shared worldview about accountability. Including more researchers working on health and accountability and aligned with other communities, such as law, organizational management, social psychology or political science, may have led to somewhat different rankings. As mentioned in the discussion above, while the 36 questions were intended to be distinct from one another and to clearly convey different domains of potential research, some questions overlapped and could be interpreted to cover the same core research need. Rankings may in some cases reflect a preference for one question’s emphasis, syntax or illustrative examples over another’s, rather than a deeper assessment of higher and lower research priority. Finally, it is important to note that Co-Digital uses a proprietary algorithm that drops low ranking questions from later voting rounds. Since exact details of when low-ranked questions dropped out of voting are not available, the ranked order of questions towards the bottom half of the list of 36 questions should be interpreted loosely.

## Conclusion

While our overview of reviews demonstrates a burgeoning field of work on accountability and participation in health, there is no scoping review that provides a systematic picture of the nature of studies funded and conducted in this field, so it is difficult to assess whether the research agenda proposed here constitutes a substantial shift from the current pattern of research in this field. The outcomes of this priority setting exercise echo earlier calls from global representatives of government, academic institutions and civil society for greater research into macro level political dimensions of accountability and on the ‘effectiveness of different regulatory, incentive, oversight, participation or decision making options for wider health systems’ (Alliance for Health Policy and Systems Research, 2008). However, our sense from the overview of reviews, and from discussions with many of the researchers engaged in this activity, is that to-date some of the largest research investments have focused on intervention studies that have sought to assess the effectiveness of specific measures to strengthen accountability. For example the ‘Making All Voices Count’ initiative, a US$45 million governance initiative—not specific to health—sought to support and research ‘innovative solutions’ with a particular focus on mobile health and digital technology (USAID, 2013; Hivos, Ushahidi, and Institute of Development Studies, 2017). Similarly, the large scale Transparency for Development Project seeks to ‘design, pilot and rigorously evaluate a series of T/A [transparency and accountability] interventions across several countries’ (Results for Development, 2018). This approach of identifying ‘interventions’ that can be incorporated into country programmes is typical of public health, but the agenda defined here reflects different priorities, instead emphasizing the need to understand the political economy of contexts, and work with existing systems of accountability.

The success of health policy and systems research depends critically on policymakers and practitioners finding research relevant to their needs. We have sought to maximize the policy-relevance and utility of research conducted and the impact of research investments through engaging such practitioners in identifying research priorities. Experts prioritized research into how context and actors shape effectiveness, the processes for implementing accountability interventions, and the effectiveness of different interventions and packages of interventions to promote accountability. Researchers firmly locate health systems accountability within larger political systems, and appear eager to grapple with how best to understand the linkages between broader contextual forces and accountability. Research on the priority questions identified here will generate knowledge on how broad system-level changes serve as enabling environments for citizen action, and bolster public sector management and responsiveness.

## Supplementary Material

Supplementary Annex 1

Supplementary Annex 2

Supplementary Annex 3

Supplementary Annex 4
