# Supplementary material for: Developing more participatory and accountable institutions for health: identifying health system research priorities for the Sustainable Development Goal-era
Source: Health Policy Plan. 2018 Sep 20;33(9):975–87. doi: 10.1093/heapol/czy079 (PMC6263024; doi:10.1093/heapol/czy079)
Supplement: Supplementary Annex 1 [file czy079_online_annex_1.docx]

## Annexure 1. Search terms for review of reviews

**Search A: Health committees**

| Two concepts: (1) reviews + (2) health committees  **Concept 1: reviews**  Pubmed  review  OR  "systematic review"  OR  "systematic literature"  OR  meta-analysis  OR  "meta analysis"  OR  metaanalysis  OR  metanalysis  Scopus  AND (review OR "systematic review" OR "systematic literature" OR meta-analysis OR "meta analysis" OR metaanalysis OR metanalysis))  **Concept 2:  health committees**  Pubmed  "village health committee*"  OR  ( "health committee*"  AND  ( community  OR  village ) )  OR   ("local committee*"  AND health) OR   "health facility committee*"  OR   "health cent* committee*"  OR   "village development committee*"  OR   "facility committee*"  OR   "health planning group"  OR   "health facility operation and management committee*"  OR   "health social action committee*"  OR   "municipal health council"  OR   panchayat  OR   ( "health board"  AND  ( village  OR  community  OR  municipal ) )  Scopus  (health OR healthcare OR "health care" OR "public health")  AND  (("village health committee" OR "village health committees" OR (("health committee" OR "health committees") AND ( community OR village )) OR ("local committee" AND health) OR "health facility committee" OR "health facility committees" OR "health centre committee" OR "health centre committees" OR "health center committee" OR "health center committees" OR "village development committee" OR "facility committees" OR "health planning group" OR "health planning groups" OR "health facility operation committee" OR "health social action committee" OR "municipal health council" OR panchayat) |
| --- |

**Search B: Score cards**

| Four concepts: (1) review + (2) health + (3) score cards + (4) local/village  **Concept 1: reviews**  [same as above]  **Concept 2: health**  Pubmed  Search word “health”  Scopus  {health} OR {healthcare}  **Concept 3: score cards**  Pubmed  "Score card"[text word] OR "scorecard"[text word] OR "score cards"[text word] OR "report card"[text word] OR "report cards"[text word] Or "check list"[text word] OR "checklist"[text word]  Scopus  {Score card} OR {scorecard} OR {score cards} OR {report card} OR {report cards} OR {check list} OR {checklist}  **Concept 4: village/local**  Pubmed  civic[text word]  OR village[text word] OR villages[text word] OR municipalities[text word] OR municipal[text word]  OR  local[text word]  OR  community[text word]  OR communities[text word] OR  participat*[text word]  OR  grassroots[text word]  OR periphery[text word]  OR  peripheral[text word] OR "Rural Health Services"[Mesh] OR "Rural Health"[Mesh] OR "rural"[text word]  Scopus  {civic}  OR {village} OR {villages} OR {municipalities} OR {municipal}  OR  {local}  OR  {community}  OR {communities} OR  {participat*}  OR  {grassroots}  OR {periphery}  OR  {peripheral} OR {rural} |
| --- |

**Search C: Local governance and accountability**

| Four concepts: (1) review + (2) health + (3) governance/accountability + (4) village/local  **Concept 1: review**  [same as above]  **Concept 2: health**  [same as above]  **Concept 3: governance/accountability**  Pubmed  (engag* OR governance OR accountability OR corruption OR nepotism OR decentralisation OR decentralization OR devolution )  Scopus  (engag* OR governance OR accountability OR corruption OR nepotism OR decentralisation OR decentralization OR devolution )  **Concept 4: local**  Pubmed  civic  OR village OR villages OR municipalities OR municipal OR local OR community OR communities OR  participat* OR  grassroots  OR periphery  OR  peripheral OR "Rural Health Services"[Mesh] OR "Rural Health"[Mesh] OR "rural"[tw]  Scopus  ( civic OR village OR villages OR municipalities OR municipal OR local OR community OR communities OR participat* OR grassroots OR periphery OR peripheral ) |
| --- |
