# Supplementary material for: Developing more participatory and accountable institutions for health: identifying health system research priorities for the Sustainable Development Goal-era
Source: Health Policy Plan. 2018 Sep 20;33(9):975–87. doi: 10.1093/heapol/czy079 (PMC6263024; doi:10.1093/heapol/czy079)
Supplement: Supplementary Annex 2 [file czy079_online_annex_2.pdf]

Annexure 2. Data fields extracted for overview of reviews

Component	Specific data extracted
**Metadata**	First Author
	Date
	Title
	Source (journal, volume, issue, page range)
	Abstract
	Source
**Overview of the review**	Type of review, according to authors: systematic review, synthesis, literature review, review of case studies, etc.
	Interventions
	Outcomes of interest
	# papers identified
	# papers from LMICs
	Regional considerations (LMIC, HIC or global)
	Primary focus
	Main research question
	Main findings
**Strategies for strengthening civic engagement and local accountability including health committees**	Describe interventions relevant to civic engagement & local accountability including health committees
	What is the evidence regarding effectiveness of the intervention?
	What findings if any are there about how the policy environment and institutional structures supported or undermined the intervention?
	What findings, if any, are there on how implementation of the intervention proceeds, and possible barriers or facilitators to the intervention?
	Other important findings
**Decentralization or devolution**	Describe interventions relevant to decentralization or devolution
	What is the evidence regarding effectiveness of the intervention?
	What findings if any are there about how the policy environment and institutional structures supported or undermined the intervention?
	What findings, if any, are there on how implementation of the intervention proceeds, and possible barriers or facilitators to the intervention?
	Other important findings
**Community score cards**	Describe interventions relevant to community score cards
	What is the evidence regarding effectiveness of the intervention?
	What findings if any are there about how the policy environment and institutional structures supported or undermined the intervention?
	What findings, if any, are there on how implementation of the intervention proceeds, and possible barriers or facilitators to the intervention?
	Other important findings
**Strategies to promote**	Describe interventions relevant to promoting transparency about the performance of local health systems

Component	Specific data extracted
**transparency about the performance of local health systems**	What is the evidence regarding effectiveness of the intervention?
	What findings if any are there about how the policy environment and institutional structures supported or undermined the intervention?
	What findings, if any, are there on how implementation of the intervention proceeds, and possible barriers or facilitators to the intervention?
	Other important findings
**Internal accountability**	Describe interventions relevant to promoting health system accountability which is internal to the health system (i.e. if does not involve outside stakeholders, does not increase transparency to those outside the system)
	What is the evidence regarding effectiveness of the intervention?
**Conclusions and reflections**	What do the authors say about the overall quality of the papers included?
	What do the authors say about the implications for future research and research priorities?
	What do the authors say about implications for policy and practice
	Data extractor comments on the quality of the review
