# Supplementary material for: Developing more participatory and accountable institutions for health: identifying health system research priorities for the Sustainable Development Goal-era
Source: Health Policy Plan. 2018 Sep 20;33(9):975–87. doi: 10.1093/heapol/czy079 (PMC6263024; doi:10.1093/heapol/czy079)
Supplement: Supplementary Annex 3 [file czy079_online_annex_3.docx]

## Annexure 3. Themes used to group research questions identified from scoping review and policymaker consultations

| **Theme** | **Description of theme** |
| --- | --- |
| 1. Evidence of effectiveness | Need empirical research that evaluates and documents the effectiveness of accountability interventions in the health sector  Need to explore impact of interventions on health outcomes not just immediate service outputs (like corruption) |
| 1. Systems and methods for determining effectiveness | Need to strengthen understanding of systems and methodologies to evaluation social accountability initiatives Need to better analyze and learn from failure |
| 1. Conceptual gaps | Need to better understand the processes and mechanisms that lead to outcomes  Need to more clearly identify who the stakeholders are  Need to overcome conceptual gaps in understanding transparency and accountability initiatives, which make comparability of the available evidence difficult  Need to better map the conceptual models through which social accountability initiatives produce outcomes (theories of change) |
| 1. Different actors and stakeholders | Need to consider accountability of private sector as well  Need to consider the role of the media |
| 1. Compare across contexts | Need to compare how social accountability initiatives work across different contexts so that evidence can be transferred elsewhere  Need to understand how different populations engage with initiatives, how accountability initiatives operate in hierarchical and heterogeneous populations |
| 1. Compare timelines | Need to better understand how social accountability initiatives work over time, especially long time horizons |
| 1. Incentives | Need to understand how incentives can align to bolster accountability |
| 1. Tools and design | Need to identify design features that make accountability tools user friendly and effective |
| 1. Role of information | Need to understand how information can improve social accountability |
| 1. Compare relative effectiveness of different interventions | Need to not just determine if interventions work, but how effective (including cost effective) they are in comparison to other interventions |
| 1. Synergies between interventions | Need to understand interactions between internal and external accountability/bottom up and top down/ community-based and facility-based accountability mechanisms |
| 1. Role of structural reforms | Need to understand how the extent of decentralization of accountability power influences community governance |
| 1. Scale up | Need to understand issues related to bringing interventions to scale |
| 1. Situating accountability in broader trajectories | Need to pay attention to history to understand broader trajectories, to the accountability of research on accountability, and to the alignment of global and national accountability reporting |
| 1. Underlying causes of corruption (at the macro, meso and micro level) | Need to understand the influences on frontline provider behaviour, including macro level influences (social forces/culture); meso (influences linked to organizational culture) and micro (individual traits) |
