# Supplementary material for: Developing more participatory and accountable institutions for health: identifying health system research priorities for the Sustainable Development Goal-era
Source: Health Policy Plan. 2018 Sep 20;33(9):975–87. doi: 10.1093/heapol/czy079 (PMC6263024; doi:10.1093/heapol/czy079)
Supplement: Supplementary Annex 4 [file czy079_online_annex_4.docx]

# Annexure 4. Full list of priority research questions from reviews and policymaker consultations

**Table A4: Full list of extracted research questions on participatory and accountable institutions for health**

| **Final Question and rank** | **Original Question** | **# Generations** | **Number of questions integrated to generate the synthesized question** | |
| --- | --- | --- | --- | --- |
|  |  |  | **From reviews** | **From policymaker consultations** |
| 1. What political factors (e.g. the discretionary power of health providers, politicization of the health system and other political factors) mediate the adoption or effectiveness of accountability initiatives (e.g. digital technology, health committees, local media or more informal citizen actions)? | What factors (e.g. the discretionary power of health workers, media) mediate the adoption or effectiveness of accountability initiatives (e.g. digital technology, health committees, local media or more informal citizen actions)? | 4 | 3 | 2 |
| 1. What processes and incentives (e.g. financial/non-financial, punitive/trust-building, learning loops, peer review) facilitate the acceptability of accountability mechanisms among frontline healthcare providers? | What processes and incentives (e.g. financial/non-financial, punitive/trust-building) facilitate the acceptability of accountability mechanisms among frontline healthcare providers? | 2 | 9 | 1 |
| 1. What reforms (e.g. decentralized budgeting, performance based financing) in the governance of national health systems are most likely to enhance provider accountability to consumers and in what contexts? | What reforms (e.g. decentralized budgeting, PBF) in the governance of national health systems are most likely to enhance provider accountability to consumers? | 2 | 2 | 3 |
| 1. What mechanisms and contextual/historical factors enable or hinder various actors (MoH officials, lay and professional health workers themselves, clients & communities, civil society, private sector, religious groups providing healthcare) to interact productively in order to improve accountability and responsiveness? | What mechanisms enable various actors (MoH, civil society, private sector, religious groups providing healthcare) to interact productively in order to improve accountability and responsiveness? | 3 | 1 | 3 |
| 1. What conditions or factors are necessary to enable accountability initiatives to address issues at the macro (e.g. political social, cultural and economic environment), meso (e.g. organizational culture, incentives), and micro (e.g. individual ethics, rationalizations) levels? | To what extent can accountability initiatives address issues at the macro (e.g. political social, cultural and economic environment), meso (e.g. organizational culture, incentives), and micro (e.g. individual ethics, rationalizations) levels? | 2 | 2 | 0 |
| 1. What are the impacts (expected and unexpected) of accountability interventions on the health workforce? (E.g. attitudes, behavior, practices, morale, decision-space, service provision, corruption, performance etc.) | What are the impacts (expected and unexpected) of accountability interventions on the health workforce? (Eg attitudes, behavior, practices, service provision, corruption etc) | 3 | 3 | 0 |
| 1. What is the impact (expected and unexpected) or effectiveness of transparency and accountability interventions on various aspects of governance and health system performance (e.g. healthcare quality, service utilization, human resource management, corruption, participatory decision-making, and citizen-state relationships within and beyond the health sector)? | What is the impact or effectiveness (expected and unexpected) of transparency and accountability interventions on various aspects of accountability, governance and/or health system performance (e.g. healthcare quality, corruption, citizen-state relationships beyond the health sector)? | 5 | 12 | 6 |
| 1. How can citizen monitoring and evaluation be effectively integrated into health system planning and implementation? | How can monitoring and evaluation be effectively integrated into health system planning and implementation? | 3 | 3 | 1 |
| 1. What tools and design features (e.g. format, frequency of use, degree of standardization) can enhance the effectiveness of accountability initiatives, such as digital reporting tools, report cards, social audit tools/social autopsy tools, community report on outbreak responses etc.? | What tools and design features (e.g. format, frequency of use, degree of standardization) can enhance the effectiveness of accountability initiatives, such as digital reporting tools, report cards, social audit tools, and social autopsy tools, community report on outbreak responses etc...? | 1 | 8 | 5 |
| 1. How do specific contexts (e.g. political environment, strength of democracy, social cohesion/heterogeneity, level of economic inequity, health system privatization) influence the potential for success/failure of particular types of accountability initiatives? | How do specific contexts (e.g. political environment, strength of democracy, social cohesion/heterogeneity, level of economic inequity, health system privitization) influence the potential for success/failure of particular types of accountability initiatives? | 1 | 8 | 4 |
| 1. What structures, processes and incentives empower (or fail to empower) citizens to engage with accountability initiatives? And for which groups of citizens? | What processes and incentives empower (or fail to empower) citizen engagement with accountability initiatives? And in what context? | 3 | 12 | 6 |
| 1. How can multiple existing data sources (e.g. from health information systems, complaints mechanisms, independent civil society monitoring) be integrated into decision making at all levels such that it more efficiently and effectively drives accountability? | How can multiple existing data sources (eg. from health information systems, complaints mechanisms, or community audits) be more efficiently and effectively used to drive accountability? | 4 | 1 | 4 |
| 1. How can citizen-driven external accountability mechanisms interact synergistically with institutionally-driven internal accountability mechanisms? | How can citizen-driven external accountability mechanisms interact synergistically with institutionally-driven internal accountability mechanisms? | 1 | 4 | 3 |
| 1. What types of information and modes of delivering information can enable citizens to respond in ways that increase accountability? Under what conditions? | What types of information and modes of delivering information will enable citizens to respond in ways that increase accountability? | 2 | 2 | 3 |
| 1. What is the effectiveness of social accountability interventions across longer timeframes (e.g. medium term and long term) and what factors enable sustainability? | What is the effectiveness of social accountability interventions across longer timeframes (e.g. medium term and long term) and what factors enable sustainability? | 2 | 5 | 0 |
| 1. How can media (i.e. traditional media, social media) be best engaged to support accountability activities? | How can media (i.e. tradition media, social media) be best engaged to support accountability activities? | 2 | 2 | 2 |
| 1. How do stakeholder interests and power dynamics between actors affect social accountability, and social accountability interventions? | How do stakeholder interests and power dynamics between actors affect social accountability, and social accountability interventions? | 1 | 2 | 2 |
| 1. What processes and incentives (e.g. enforcement, changes in international/domestic environment, financial investments, etc.) facilitate the acceptability of accountability mechanisms among senior policy makers and/or political elites? | What processes and incentives (e.g. enforcement, changes in international/domestic environment, financial investments, etc.) facilitate the acceptability of accountability mechanisms among senior policy makers and/or political elites? | 1 | 7 | 0 |
| 1. What is the impact of decentralization on accountability? (E.g. at the various government levels, with changing roles and responsibilities etc.)? | What is the impact of decentralization on accountability? (E.g. at the various government levels, with changing roles and responsibilities etc.)? | 1 | 3 | 4 |
| 1. What are the theoretical models underpinning various accountability processes as well as interventions (e.g. how are they supposed to work, what are they supposed to impact, who is supposed to be engaged)? What assumptions (implicit and/or explicit) do these models make? | What are the theoretical models underpinning various accountability processes as well as interventions (e.g. how are they supposed to work, what are they supposed to impact, who is supposed to be engaged)? | 2 | 6 | 0 |
| 1. How does information-for-accountability diffuse among citizens and civil society's social networks? | How does information-for-accountability diffuse among citizens’ social networks? | 2 | 1 | 1 |
| 1. How can we align global mechanisms/initiatives for accountability (e.g. global reporting on MCH goals), with local or country-led reporting? | How can we align global mechanisms/initiatives for accountability e.g. global reporting on MCH goals, with local or country-led reporting? | 1 | 0 | 1 |
| 1. How and why do social accountability interventions by different types of organizations (e.g. NGO vs. non-NGO) compare? (E.g. effectiveness, sustainability and potential for mainstreaming) | How and why do social accountability interventions by different types of organisations (e.g. NGO vs. non-NGO) compare? (e.g. effectiveness, sustainability and potential for mainstreaming?) | 3 | 1 | 1 |
| 1. In what ways does the healthcare organization’s environment (e.g. its values, mission, and culture, or other factors such as contract design, allowance for managerial discretion, clinical governance, processes, and procedures) affect its approach to accountability, and how transferable are these feature to other organizations? | In what ways does the healthcare organization's environment (e.g. its values, mission, and culture, or other factors such as contract design) affect its approach to accountability, and how transferable are these feature to other organizations? | 3 | 1 | 0 |
| 1. How do social accountability mechanisms targeting different types of healthcare providers (government, private for-profit, private not-for-profit, public-private partnership) differ (e.g. in their methods or effectiveness)? | How do social accountability mechanisms compare when applied to different types of healthcare provider models (government, private for-profit, private not-for-profit, PPP)? | 3 | 1 | 2 |
| 1. What can different research paradigms and methodologies (e.g. mixed methods, realist evaluation, institutional ethnography, institutional change theory, power analysis, complex interventions, participatory research, interactive learning and action approach, social network analysis) contribute to the assessment of social accountability initiatives? | What can different research methodologies (mixed methods, path dependency, institutional change theory, power analysis) and disciplines contribute to the assessment of social accountability initiatives? | 5 | 3 | 1 |
| 1. What is the potential role of information and communication technology on social accountability? (e.g. innovative ways to collect information about accountability in real time, mobilize communities through social media, overcome barriers to vulnerability, reduce risk and facilitate collective action) | What is the impact of digital technology initiatives on social accountability? (e.g. innovative ways to collect information about accountability in real time, mobilize communities through social media, etc.) | 4 | 3 | 2 |
| 1. What factors affect the scale-ability of accountability interventions? What do programs at scale look like? | What factors affect the scale up of accountability interventions? | 3 | 2 | 2 |
| 1. How do we define social accountability in the context of health (boundaries, constructs, core elements etc.)? What can we draw from discourse in other fields (political science, organizational sociology, philosophy and social psychology)? | How do we define social accountability in the context of health (boundaries, constructs, core elements etc.)? What can we draw from discourse in other fields (political science, organizational sociology, philosophy and social psychology?) | 1 | 3 | 2 |
| 1. In what way do gender relations (e.g. in government/bureaucratic positions, in community monitoring groups) influence the success of social accountability initiatives? | In what ways does gender (e.g. representation of women in government/bureaucratic positions, role of women in community monitoring groups) influence the success of social accountability initiatives? | 1 | 1 | 0 |
| 1. What context specific factors trigger accountability initiatives? | What context specific factors trigger accountability activities? | 2 | 2 | 0 |
| 1. What are the impacts (expected and unexpected) of transparency and accountability interventions on health and social outcomes (e.g. social cohesion, equity, empowerment, trust)? | What are the impacts (expected and unexpected) of accountability initiatives on health outcomes? | 5 | 5 | 4 |
| 1. What were the circumstances/factors that led to accountability interventions failing to produce desired results? | What were the circumstances/factors that led to accountability interventions failing to produce desired results? | 1 | 1 | 0 |
| 1. How can researchers studying and intervening in health system accountability be themselves accountable to the communities and the health systems they seek to improve? | How can researchers studying health system accountability be themselves accountable to societies? | 2 | 1 | 1 |
| 1. How do we institute accountability in contexts affected by conflict (e.g. internally displaced populations, refugees, etc.)? | How do we institute accountability in contexts affected by conflict (Internally displaced populations, refugees, etc.)? | 1 | 0 | 1 |
| 1. What is the cost effectiveness of different accountability interventions versus other forms of oversight and governance? | What is the cost effectiveness of different accountability interventions versus other forms of oversight and governance? | 1 | 4 | 3 |
